# Integrative analyses of genes related to femoral head osteonecrosis: an umbrella review of systematic reviews and meta-analyses of observational studies

**DOI:** 10.1186/s13018-022-03079-4

**Published:** 2022-03-28

**Authors:** Sangyeob Lee, Jun-Il Yoo, Yang-Jae Kang

**Affiliations:** 1 Department of Biomedical Research Institute, Gyeongsang National University Hospital, Jinju, Republic of Korea; 2 Department of Theriogenology and Biotechnology, College of Veterinary Medicine, Gyeongsang National University, Jinju, Republic of Korea; 3 Department of Orthopaedic Surgery, Gyeongsang National University Hospital and College of Medicine, 90 Chilamdong, Jinju, Gyeongnamdo 660-702 Republic of Korea; 4 Division of Life Science Department, Gyeongsang National University, Jinju, Republic of Korea

**Keywords:** Femoral head osteonecrosis, Umbrella review, Steroid, Polymorphism, Genetic variant

## Abstract

**Background:**

Femoral head osteonecrosis (FHON) is a worldwide challenging clinical topic. Steroid use is one of the main etiologies of FHON. There are several genetic variants associated with FHON. Therefore, the purpose of this umbrella review was to provide a comprehensive summary of a meta-analysis and systematic review of genetic variations associated with nonsteroidal and steroid-induced FHON.

**Methods:**

The eligible studies were selected from the PubMed and MEDLINE databases for the collection of diverse systematic meta-analyses and reviews. The genetic main effect score was assigned using the Human Genome Epidemiology Network’s Venice criteria to assess the cumulative evidence on the effects of a single nucleotide polymorphism (SNP) on FHON.

**Results:**

Eight articles reported the meta-analysis of candidate SNP-based studies covering eight genes and 13 genetic variants. In the nonsteroid-induced FHON genetic variants including rs2012390 and rs11225394 in MMP8, rs1800629 and rs361525 in tumor necrosis factor (TNF)-α, VNTR in intron 4, rs1799983 and rs2070744 in endothelial nitric oxide synthase (eNOS), rs2010963 in vascular endothelial growth factor (VEGF), and rs6025 in factor V showed significance in each reference. The steroid-induced FHON genetic variants including rs693 and rs1042031 in apolipoprotein (Apo)B, rs1045642 in ABCB1, and rs1799889 in PAI-1 showed significance in each reference.

**Conclusion:**

Based on the systematic review conducted in this study, we organized the genomes associated with FHON and looked at each contribution. Our results could give an integrative approach for understanding the mechanism of FHON etiology. It is expected that these results could contribute to the strategy of prediagnosis, evaluating the individual risk of nonsteroid-induced and steroid-induced FHON.

*Level of Evidence*: Level I.

**Supplementary Information:**

The online version contains supplementary material available at 10.1186/s13018-022-03079-4.

## Introduction

Femoral head osteonecrosis (FHON) is a worldwide challenging clinical topic. FHON was described as hip trauma with late complications by Jean Cruveilhier, a French anatomist and pathologist [1]. Since then, many studies have identified the etiology of FHON [1–4]. According to studies that conducted pathology of FHON, classification of pathogenesis included the following. First, obstructed circulation to a specific area due to compromised blood flow was reported as the final pathway of FHON. Second, steroid-induced FHON has different pathogenesis, which is a kind of intraosseous compartment syndrome. Third, FHON has a complicated etiology including genetic factors and exposure to risk factors. This means that genetic factors can be the cause of disease emergence and are closely related to other pathogenesis factors [5–8]. The risk of FHOM increases in the presence of these genetic predisposing factors [9].

Steroid use is one of the main etiologies of FHON. Steroids are dose-dependent etiologic agents of FHON [10]. Long-term steroid use could lead to the collapse of the femoral head, structural alteration, and dysfunctional hip joint [11]. There are several pathogenesis theories of steroid-induced FHON, including (1) the lipid metabolism disorder theory that steroids cause fat embolism within the microvessels by increasing subcutaneous fat mobilization [12]; (2) the insufficient blood supply theory that some steroids disturb the blood supply to the femoral head with intravascular thrombosis [13, 14]; and (3) adipogenesis of the bone marrow stromal cells (BMSCs), which is the principal mechanism involved in the onset and progression of steroid-induced FHON [15, 16]. Although many theories are considered the etiology of steroid-induced FHON, the exact pathologic process is still not clear.

So far, several genetic factors associated with FHON have been reported including apolipoprotein (Apo)A, which is involved in lipid metabolism and the coagulation system [17, 18]; VEGF in the Korean population [19]; mutations in factor V Leiden (FVL) [20]; and plasminogen activator inhibitor type 1 polymorphism [21, 22]. In addition, genetic factors associated with steroid-induced FHON have also been reported including genes related to lipid transportation like ABCB1and CYP3A [23, 24]; and genetic polymorphisms involved in vascular occlusion and coagulopathy such as PAI and MTHFR [25–27]. These genetic factors are very useful tools for understanding FHON. Identifying the specific genetic differences between those with and without FHON will provide clues to FHON.

Therefore, the purpose of this umbrella review was to provide a comprehensive summary of a meta-analysis and systematic review of genetic variations associated with nonsteroidal and steroid-induced FHON.

## Methods

### Search strategy and eligible study selection criteria

The eligible studies were selected from the PubMed and MEDLINE databases for the collection of diverse systematic meta-analyses and reviews in accordance with Preferred Reporting Items for Systematic Reviews and Meta-Analyses guidelines [28]. The search strategy included the keywords "osteonecrosis"[MeSH Terms] OR "osteonecrosis"[All Fields] OR "avascular"[All Fields] AND "necrosis"[All Fields] OR "avascular necrosis"[All Fields]) AND "genes"[MeSH Terms] OR "genes"[All Fields] OR "gene"[All Fields] AND "meta"[Journal] OR "meta"[All Fields]. The retrieved publications were independently screened by two authors. Discrepancies were resolved according to a consensus. A third investigator was included if a final decision could not be reached. After the title and abstract were screened, the full texts of the publications were selected for determining the final eligibility.

The eligible studies included meta-analyses of four types: (i) SNP-based systematic reviews and gene meta-analyses related to femoral neck head necrosis, (ii) studies providing definite information on the statistical processes and results, and (iii) studies written in English and published after January 1, 2012. Only studies in adult populations were included, and gender differences were not considered because general SNP-based meta-analyses associated with FHON were performed regardless of sex. The following types of studies were excluded: (i) systematic meta-analyses without a quantitative synthesis of the evidence, (ii) non-human studies, (iii) having huge errors or poor quality, and (iv) studies with no specific statistical results including heterogeneity tests, the examined risk factors, and the overall *p* values.

### Data extraction

Two independent authors were included in the data extraction stage. One author extracted the data and then it was checked by a second author. For selecting the eligible articles, the following information was included: (i) the first author’s name; (ii) year of publication; (iii) the genetic variants of each study and effect size (ES); (iv) the number of studies included in each article; and (v) sample size, *p* values, and heterogeneity estimates.

### Methodological quality

Methodological quality was rated by the Assessment of Multiple Systematic Reviews (AMSTAR) instrument [2]. This version comprises 16 items evaluating the methodological quality of articles. This assessment is rated on a scale from high quality to very low quality.

### Statistical analysis

The genetic main effect score was assigned using the Human Genome Epidemiology Network’s Venice criteria to assess the cumulative evidence of SNP effects on FHON [29]. Briefly, these guidelines provide criteria for assessing cumulative evidence in genetic epidemiology. The amount of evidence, the extent of replication, and protection from bias were indexes in these guidelines. The three levels of evidence were strong, moderate, or weak. According to these criteria, a large amount of evidence and large sample sizes ensure adequate power for detecting an association. By the genome-wide testing of thousands of polymorphisms, we evaluated the evidence for the main genetic effect score class with *p* values of at least < 0.005 to be considered for strong candidacy. A specific description of the application of the guidelines is provided in Table 1.

**Table 1:** Description of the extension of the Human Genome Epidemiology Network’s Venice criteria to assess genetic main effect

Criteria of consideration	Category	Proposed operationalization
Amount of evidence	A	Sample size over 1000
	B	Sample size 100–1000
	C	Sample size under 100
Replication	A	*I*^2^ < 50%
	B	25% < *I*^2^ < 50%
	C	*I*^2^ > 50%
Protection from bias	A	Consideration biases such as bias in genotyping, population stratification, and Selective reporting biases
	B	Bias in genotype definition = Not reported what was done/No quality control checks/Appropriate quality control checks
	C	Population stratification = Not reported what was done/Nothing done/Same descent group/Adjustment for reported descent/Family-based design/Genomic control, PCA or similar method Selective reporting biases = Meta-analysis of published data/Retrospective efforts to include unpublished data/Meta-analysis within consortium

Evidence is classified as strong, moderate, and weak. When scored A in every criteria, evidence is categorized as strong. When scored no C in every criteria but no AAA, evidence is categorized as moderate. Weak evidence is recorded with C in one out of three criteria

## Results

### Number of articles and type identified

**Fig. 1:**
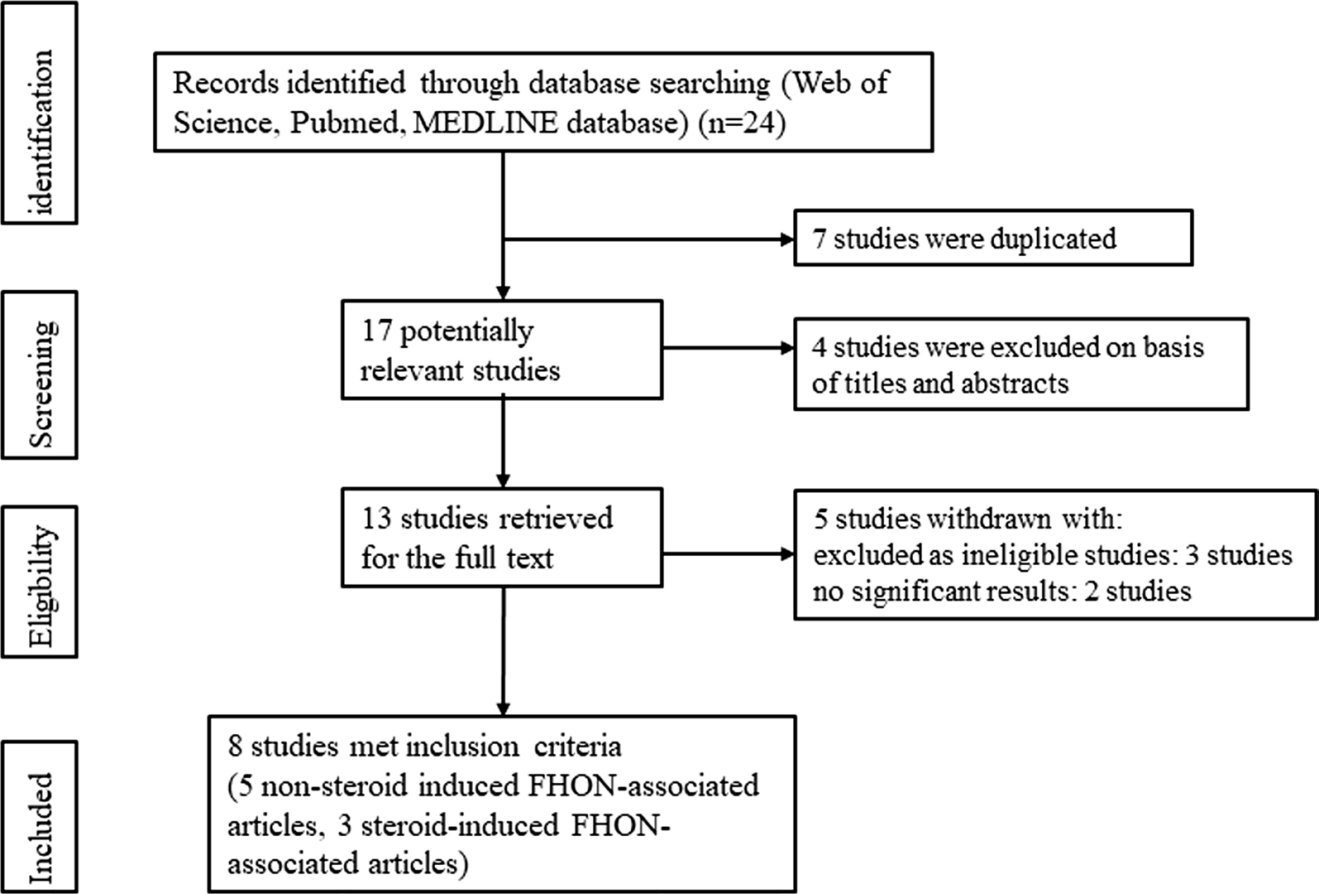
Flowchart of study selection process

For the systematic reviews, 24 unique references were identified from the databases. After excluding the duplicates and articles that were not on the main topics, 13 articles remained. Three additional articles were excluded as ineligible studies because they provided inadequate data. Two other articles with no significant results were excluded. Finally, eight eligible publications [30–37] were selected for references as they included convincing data, appropriate qualitative evaluations, significant results, human studies, and topics fitting with our purpose (Fig. 1). In the eight included references, there were five nonsteroid-induced FHON-associated articles including nine genetic variants and three steroid-induced FHON-associated articles including four genetic variants. All references were retrospective studies to identify FHON-associated genetic variants. And all studies included in the references used blood samples for subject-specific genotyping. The publications that excluded with various reasons were provided in Additional file 1: Table S1.

### Main findings of the meta-analyses of SNP-based studies

Eight articles reported the meta-analyses of candidate SNP-based studies covering eight genes and 13 genetic variants.

In nonsteroid-induced FHON, MMP-8 displayed meaningful results in rs2012390 and rs11225394. Rs11225394 was very significant, with a *p* value of under 0.00001 for the genetic main effect. The rs11225394 SNP showed a strong evidence class according to the Venice criteria. Peng et al. [31] demonstrated that rs1800629 and rs361525, which are SNPs of TNF-α, showed a low odds ratio with the G allele. In rs361525, the odds ratio was under 0.3 and the *p* value was under 0.001 in the allele genetic model and recessive genetic model, respectively. This result of rs361525 showed extremely significant and strong evidence with a score of AAB in the Venice criteria. Song and Lee et al. [32] reported that the variable number of tandem repeats (VNTRs) in intron 4 of the eNOS gene showed high significance with a *p* value of under 0.001 in the allele genetic model and the dominant genetic model. The evidence class was moderate. Rs1799983 and rs2070744 also showed barely significant results and excessive heterogeneity with *I*^2^ values of 80.20% and 90%, respectively. Rs2010963 of the VEGF gene in Asians also showed significant results in the homozygote model and the dominant model with strong evidence [33]. A *p* value of < 0.001 with strong evidence was recorded in rs6025, which is a genetic variant of factor. Shang et al. demonstrated that rs6025 in a mixed ethnicity population showed a high possibility of disease occurrence with an odds ratio of 4.55 (Table 2).

**Table 2:** Genetic variants derived from eligible meta-analyses of nonsteroid-induced FHON

Genetic variant	Gene (or near gene)	Reference (year of data collection)	Ethnicity	Sample size (case/control)	Included studies	Minor allele & Reference allele	Genetic model	Type of model	Reported OR (95% CI)	*p* value for genetic main effect	Heterogeneity (%)	Venice criteria	Evidence class
rs2012390	MMP-8	Jiang et al. [30]	Asian	884/1012	3	A & G	Allele model	Fixed effect	**1.34 [1.00–1.79]**	**0.005**	**0**	NA	NA
							Heterozygote model	Fixed effect	**1.40 [1.14–1.73]**	**0.002**	**0**	NA	NA
							Dominant model	Fixed effect	**1.39 [1.13–1.71]**	**0.002**	**0**	NA	NA
rs11225394	MMP-8	Jiang et al. [30]	Asian	1184/1211	3	C & T	Heterozygote model	Fixed effect	**0.63 [0.51–0.77]**	** < 0.00001**	**21**	AAB (equivalent to AAA)	Strong
rs1800629	TNF-α	Peng et al. [31]	Caucasian/Asian	432/760	7	G & A	Allele model	Fixed effect	**0.73 [0.57–0.93]**	**0.012**	**17.50**	NA	NA
							Homozygote model	Fixed effect	0.52 [0.26–1.05]	0.069	10.00	NA	NA
							Heterozygote model	Fixed effect	0.68	0.324	6.60	NA	NA
							Dominant model	Fixed effect	0.54 [0.27–1.09]	0.085	10.40	NA	NA
							Recessive model	Fixed effect	**0.73 [0.54–0.97]**	**0.029**	**0**	NA	NA
rs361525	TNF-α	Peng et al. [31]	Caucasian/Asian	432/760	3	G & A	Allele model	Fixed effect	**0.27 [0.15–0.49]**	** < 0.001**	**0**	AAB (equivalent to AAA)	Strong
							Recessive model	Fixed effect	**0.25 [0.14–0.47]**	** < 0.001**	**0**	AAB (equivalent to AAA)	Strong
VNTR in intron 4	eNOS	Song and Lee [32]	Caucasian/Asian	296/329	3	4a & 4b	Allele model	Fixed effect	**3.24 [2.04–5.15]**	** < 0.001**	**0**	BAB	Moderate
							Dominant model	Fixed effect	**3.50 [2.12–5.78]**	** < 0.001**	**0**	BAB	Moderate
				193/226	2	4a & 4b	Recessive model	Fixed effect	4.76 [0.77–59.61]	0.094	0	NA	NA
rs1799983	eNOS	Song and Lee [32]	Asian	403/661	3	T & G	Allele model	Random effect	**1.72 [0.80–3.70]**	**0.006**	**80.20**	ABB	Moderate
rs2070744	eNOS	Song and Lee [32]	Asian/NA	145/378	2	C & T	Allele model	Random effect	**1.03 [0.19–5.96]**	**0.001**	**90**	ABB	Moderate
rs2010963	VEGF	Hong et al. [33]	Asian	697/877	3	C & G	Homozygote model	Fixed effect	**0.82 [0.72–0.93]**	**0.002**	**0**	AAB (equivalent to AAA)	Strong
							Dominant model	Fixed effect	**0.79 [0.67–0.92]**	**0.003**	**0**	AAB (equivalent to AAA)	Strong
							Recessive model	Fixed effect	**1.29 [1.06–1.59]**	**0.015**	**0**	NA	NA
rs6025	Factor V	Shang et al. [34]	Mixed	481/867	7	A & G	Homozygote model	Fixed effect	**4.55 [2.75–7.52]**	** < 0.001**	**0**	AAB (equivalent to AAA)	Strong

The signature of bold represents OR,* p* value, and heterogeneity of genetic variances with a* p* value < 0.05

Evidence is classified as strong, moderate, and weak. When scored A in every criteria, evidence is categorized as strong. When scored no C in every criteria but no AAA, evidence is categorized as moderate. Weak evidence is recorded with C in one out of three criteria

In steroid-induced FHON, ApoB has genetic variants rs693 and rs1042031 with significant *p* values for the main effect [35]. Particularly, rs1042031 showed a very significant result in the dominant genetic model with an odds ratio of 2.90 in various ethnicities and an odds ratio of 4.81 in Asians. In the articles by Zhou et al. [36], the ABCB1 rs1045642 genetic variant also showed significant results in Asians. The PAI-1 rs1799889 genetic variant showed an exceedingly significant *p* value of under 0.0005 in the homozygote model and dominant model, with a moderate evidence class in the study by Gong et al. [37].

The 16 excluded publications and the reasons are shown in Table 3.

**Table 3:** Genetic variants derived from eligible meta-analyses of steroid-induced FHON

Genetic variant	Gene (or near gene)	Reference (year of data collection)	Ethnicity	Sample size (case/control)	Included studies	Minor allele & major allele	Genetic model	Type of model	Reported OR (95% CI)	*p* value for genetic main effect	Heterogeneity (%)	Venice criteria	Evidence class
rs693	ApoB	Chen et al. [35]	Mixed	570 (total)	4	C & T	Allelic model	NA	2.63 [0.92–7.53]	0.072	58.00	NA	NA
							Heterozygous model	NA	**2.46 [1.27–4.77]**	**0.008**	**54.50**	NA	NA
							Homozygous model	NA	**7.70 [1.23–48.18]**	**0.029**	**24.40**	NA	NA
							Recessive model	NA	**7.16 [1.19–43.05]**	**0.031**	**32.10**	NA	NA
rs693	ApoB	Chen et al. [35]	Mixed	725 (total)	5	C & T	Dominant model	NA	**2.99 [1.71–5.22]**	** < 0.001**	**31.30**	BBB	Moderate
rs1042031	ApoB	Chen et al. [35]	Mixed	572 (total)	4	G & A/C	Dominant model	NA	**2.90 [1.49–5.65]**	**0.002**	**50.30**	BCB	Weak
rs1042031	ApoB	Chen et al. [35]	China	415 (total)	3	G & A/C	Dominant model	NA	**4.81 [2.05–11.30]**	** < 0.001**	**0**	BAB	Moderate
rs1801133	MTHFR	Chen et al. [35]	Mixed	251 (total)	3	C & T	Allelic	NA	0.92 [0.59–1.44]	0.710	12.90	NA	NA
							Heterozygous model	NA	0.62 [0.33–1.17]	0.144	22.10	NA	NA
							Homozygous model	NA	1.24 [0.48–3.21]	0.653	0	NA	NA
							Recessive model	NA	1.54 [0.63–3.76]	0.339	0	NA	NA
				507 (total)	4	C & T	Dominant model	NA	0.94 [0.61–1.45]	0.775	23.40	NA	NA
rs1045642	ABCB1	Zhou et al. [36]	Asian/mixed	336/712	7	T & C	Allelic	Fixed effect	0.68 [0.54–0.84]	0.295	18.30	NA	NA
							Heterozygous model	Fixed effect	0.73 [0.53–1.00]	0.722	0	NA	NA
							Homozygote model	Random effect	**0.43 [0.26–0.69]**	**0.049**	**58.10**	NA	NA
							Recessive model	Random effect	**0.52 [0.34–0.81]**	**0.009**	**70.30**	NA	NA
							Dominant model	Fixed effect	0.64 [0.48–0.87]	0.830	0	NA	NA
rs2032582	ABCB1	Zhou et al. [36]	Mixed	275/574	5	T/A &G	Allelic	Fixed effect	0.73 [0.58–0.90]	0.587	0	NA	NA
							Heterozygous model	Fixed effect	0.66 [0.45–0.96]	0.985	0	NA	NA
							Homozygote model	Fixed effect	0.52 [0.34–0.82]	0.560	0	NA	NA
							Recessive model	Fixed effect	0.71 [0.49–1.01]	0.385	3.9	NA	NA
							Dominant model	Fixed effect	0.61 [0.43–0.87]	0.959	0	NA	NA
rs1799889	PAI-1	Gong et al. [37]	Mixed	108/917	4	4G/5G	Allele model	NA	**1.93 [1.15–3.26]**	**0.014**	**0.0004 (P)**	NA	NA
							Homozygote model	NA	**3.22 [1.67–6.21]**	** < 0.0005**	**0.00111 (P)**	ACB	Moderate
							Dominant model	NA	**2.31 [1.53–3.49]**	** < 0.0005**	**0.06 (P)**	ACB	Moderate
							Recessive model	NA	**2.30 [1.24–4.30]**	**0.009**	**0.162 (P)**	NA	NA

The signature of bold represents OR,* p* value, and heterogeneity of genetic variances with a* p* value < 0.05

Evidence is classified as strong, moderate, and weak. When scored A in every criteria, evidence is categorized as strong. When scored no C in every criteria but no AAA, evidence is categorized as moderate. Weak evidence is recorded with C in one out of three criteria

### Qualitative methodological appraisal of eligible meta-analyses

The qualitative methodological appraisal was conducted using the AMSTAR tool as described in Table 4. Song et al. showed the lowest score of 7, whereas Yu Zhang et al. showed the highest score of 10.5.

**Table 4:** Qualitative methodological appraisal of eligible meta-analyses using AMSTAR tool

References	Checklist																Total
	1	2	3	4	5	6	7	8	9	10	11	12	13	14	15	16	
Chen et al. [35]	1	0.5	1	0.5	1	1	0	0.5	0	1	0	0	0	1	1	1	9.5
Jiang et al. [30]	1	0.5	1	0.5	1	1	0	0.5	0	0	0	0	0	1	1	1	8.5
Peng et al. [31]	1	0.5	1	0.5	1	1	0	0.5	0	1	0	0	0	1	1	1	9.5
Song et al., [32]	1	0.5	1	0	0	0	0	0.5	0	1	0	0	0	1	1	1	7
Hong et al. [33]	1	0.5	1	0.5	1	1	0	0.5	0	1	0	0	0	1	1	1	9.5
Zhou et al. [36]	1	0.5	1	0	1	1	0	0.5	0	1	0	0	0	1	1	1	9
Shang et al. [34]	1	0.5	1	0.5	1	1	0	0.5	0	0	0	0	0	1	1	0	7.5
Gong et al. [37]	1	0.5	1	0.5	1	1	0	0.5	0	0	0	0	0	1	1	1	8.5

Abbreviations: 0, No; 1, Yes; 1.5, Partial Yes; AMSTAR, A Measurement Tool to Assess Systematic Reviews

## Discussions

In the present study, the genes with variants in nonsteroid-induced FHON were MMP-8 (rs2012390 and rs11225394), TNF-α (rs1800629 and rs361525), eNOS (VNTR in intron 4, rs1799983, and rs2070744), VEGF (rs2010963), and factor V (rs6025). Among them, the variants in MMP8, TNF-α, eNOS, and factor V showed high significance, and particularly the variants in TNF-α (rs361525) and eNOS (VNTR in intron 4) had more than two types of model significance. Meanwhile, the genes with genetic variants in steroid-induced FHON were ApoB (rs693 and rs1042031), ABCB1 (rs1045642), and PAI-1 (rs1799889). Among them, the variants included in ApoB and PAI-1 showed high significance and particularly the variant in PAI-1 (rs1799889) had more than two types of model significance.

The PAI-1 gene is an important regulator of fibrinolysis because it plays a role as an inhibitor of plasminogen activators [38]. Rs1799889 is the most commonly studied polymorphism in the PAI-1 gene, which has a guanine deletion at the 675 nucleotide position. The PAI-1-675 4G allele shows higher transcriptional activity than the PAI-1-675 5G allele and the PAI-1-675 4G allele is related to higher PAI-1 levels in plasma [39]. In this study, the allele and recessive models of rs1799889 showed a highly significant association with steroid-induced FHON. That is, carriers of the 4G variant of PAI-1 had a higher risk of steroid-induced FHON than those with the 5G variant. It seems that the 4G allele is an important pathogenic factor with a fundamental function of disturbing PAI-1 gene expression. The low expression of the PAI-1 gene could be a cause of FHON with low fibrinolysis.

MMP-8, which is usually expressed in neutrophils but also in chondrocytes and synovial fibroblasts [40], is located on chromosome 11q22.3 [41]. The exact mechanism of the association between MMP-8 and FHON is unclear, but several studies suggested that it was the result of regulatory effects on the breakdown pathway of the extracellular matrix in bone tissue remodeling and development or that MMP-8 could affect femoral head impairment and inflammation [42, 43]. In this study, the heterozygote model of rs11225394 had a strong evidence class and high significance with a *p* value < 0.00001 and a mean OR of 0.63 in nonsteroid-induced FHON. It is supposed that MMP-8 has a protective effect in nonsteroid-induced FHON.

It is known that TNF-α promotor polymorphisms like TNF-α gene-308 or -238 affect transcriptional activity [44, 45]. Some studies demonstrated that the pathophysiology of nonsteroid-induced FHON was associated with the apoptosis of osteocytes and osteoblasts [31, 46]. In addition, TNF-α was reported to affect osteocytes and osteoblasts to release cytokines related to the proliferation and maturation of osteoclasts [47]. Therefore, polymorphisms in TNF-α could change the activation of osteoblasts and osteoclasts, which would result in the deterioration of nonsteroid-induced FHON. In this study, rs1045642, which is a polymorphism of TNF-α, showed high significance in the allele and recessive models. The mean OR was 0.27, 0.25 in the allele and recessive models, which could mean that the downregulation of TNF-α has a protective effect against nonsteroid-induced FHON.

The ABCB1 gene encodes the transport protein, p-glycoprotein (P-gp), which has crucial functions in pumping foreign substances out of cells [48]. In the study by Ning Han et al., the excessive adipogenesis of bone marrow-derived mesenchymal stem/stromal cells (BMSC) was found when P-gp was inhibited in steroid-induced FHON, indicating that bone formation in the femoral head was inhibited due to decreased BMSCs, which are precursors for osteoblast maturation [49]. In this study, rs1045642 in steroid-induced FHON decreased the risk of disease incidence with a minor T allele. It means that the ABCB1 gene might be an important gene in the regulation of steroid-induced FHON.

The eNOS gene which is located on chromosome 7q35-q36 is known to increase NO [32]. NO is known to mediate angiogenesis and vasorelaxation. However, its key role is in preventing thrombosis, which is the main pathophysiology of nonsteroid-induced FHON [50]. The 27-bp variable VNTR in intron 4 is a polymorphism of eNOS, which includes the wild 4b allele and the mutated 4a allele. This 4b/a polymorphism is related to reduced NO plasma concentrations [51]. In this study, 4b/a polymorphism in the eNOS gene showed high significance in nonsteroid-induced FHON. This might mean that the decreased expression of eNOS could lead to the incidence of nonsteroid-induced FHON. In addition, rs1799983 and rs2070744 polymorphisms in the eNOS gene also showed significance but their heterogeneity was too high (80.20% in rs1799983 and 90% in rs2070744).

VEGF is known to be highly expressed in the necrotic area of FHON. Its major role is in angiogenesis and osteogenesis [52]. The VEGF gene has also been reported to promote bone marrow cells and near endothelial cell proliferation [14, 53]. In this study, rs2010963, known as VEGF -634G/C polymorphisms, showed high significance. When all the genetic models were evaluated, the homozygote and dominant models showed a mean OR of under 1.00, while the recessive model showed a mean OR of over 1.00, suggesting that the genetic models of rs2010963 may have an important role in predicting the risk for FHON.

Factor V (FV) is well known as a component involved in the blood coagulation process [54]. The factor V Leiden (FVL) gene mutation is a typical polymorphism of FV causing thrombophilia and intravascular coagulation disorders [55]. Several studies suggested that thrombosis caused by FVL gene mutation could be the main etiology of nonsteroid-induced FHON [20, 56, 57]. In this study, rs6025, which is a polymorphism of the FVL gene, showed high significance in the homozygote model. The mean OR was 4.55. with strong evidence. This could mean that the downregulation of factor V could increase the risk of FHON occurrence.

APO is the main blood plasma protein mediating normal lipid metabolism by interacting with cellular receptors [58]. It has been reported that APOs are sensitive markers for evaluating lipid metabolic disorders in steroid-induced FHON [17, 59]. In a study by Karami et al., the C allele of rs693 had a high association with high familial cholesterol levels. In contrast, the presence of the T allele had protective effects [60]. Rs1042031 is also known as important for regulating the ApoB binding capacity to cholesterol receptors [35]. In this study, rs693 and rs1042031, which are polymorphisms of ApoB, each showed high significance in the dominant models. Their mean ORs were over 2.5, which means that if ApoB did not function well, the risk of FHON incidence could be increased. These results indicated that lipid metabolism disorders could lead to adipogenesis of the femoral head, which is one of the main causes of steroid-induced FHON [35].

**Fig. 2:**
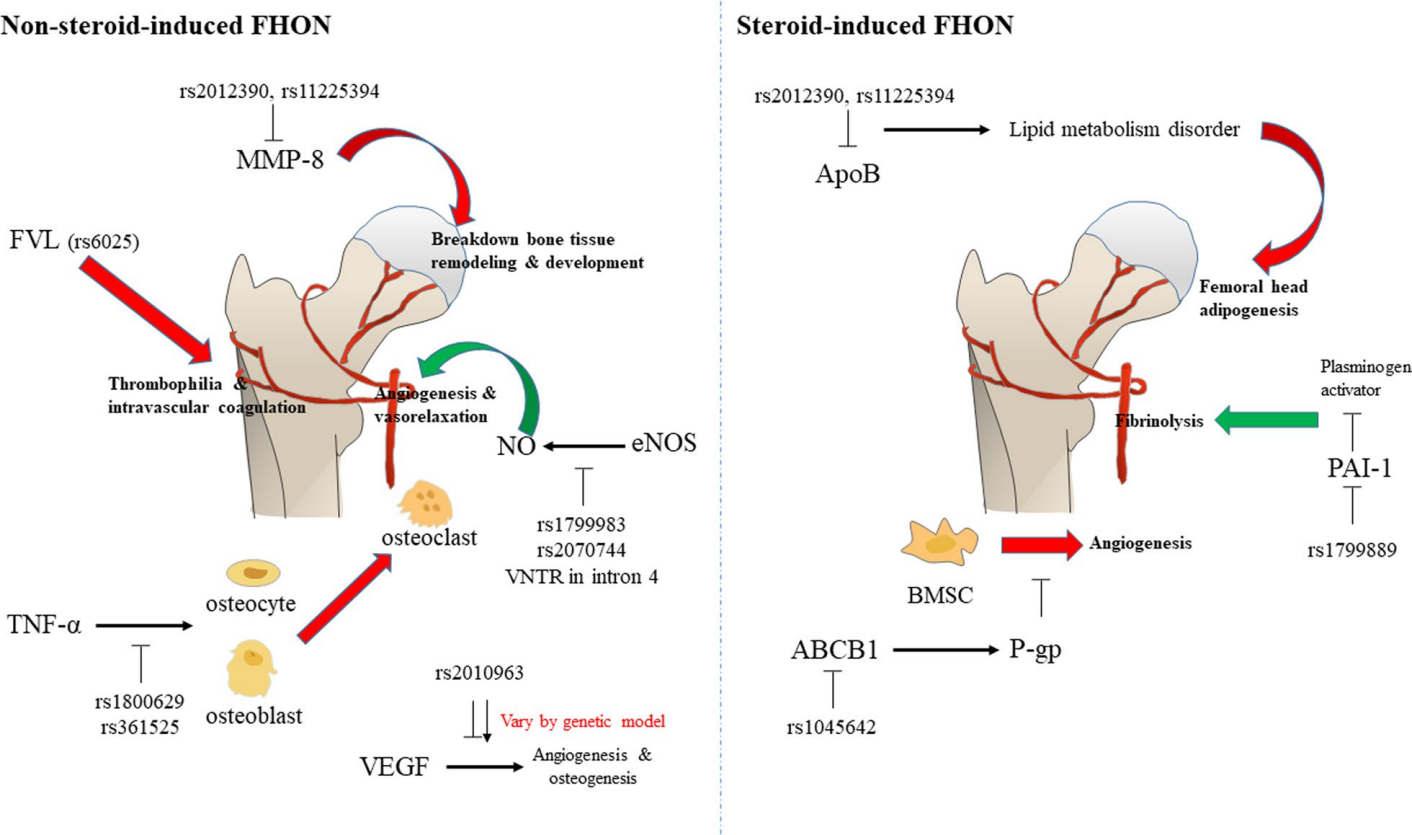
The overall gene mechanisms related to femoral head osteonecrosis (FHON). Red arrow indicates risk factors of FHON, and green arrow indicates protective factors of FHON

In brief, the nonsteroid-induced associated genes were MMP-8, TNF-α, eNOS, VEGF, and factor V, and the steroid-induced associated genes were ApoB, ABCB1, and PAI-1. The overall gene mechanisms of FHON are presented in Fig. 2.

### Limitations

The present study had several limitations. First, our study excluded gender factors in the data collection. Second, classifications of ethnicity were not specified. Third, further validation of the study results was not conducted. Fourth, although this study included many SNP results from various papers, it was limited by the studies available at the time. Fifth, this study contains a significant amount of meta-analyses derived from various references. Therefore, results might lead to an over-estimation of one or the other genes’ actual significance. Finally, most genetic variations reported in meta-analyses only appear once when searching meta-analyses containing genetic variations to collect only reliable data related to steroid-induced FHON and nonsteroid-induced FHON. This result is thought to be because there were not many meta-analyses selected for the subject of this study, and the genetic variation that each study focuses on is different.

## Conclusion

Based on a systematic review of published papers, we organized the genomes associated with FHON and examined each contribution. Our results could give an integrative approach for understanding the mechanism of FHON etiology. It is expected that these results could contribute to the strategy of prediagnosis and evaluating the individual risk of nonsteroid-induced and steroid-induced FHON.

## Supplementary Information

**Additional file 1. Table S1**: Excluded publications with reasons.

## Data Availability

Not applicable.
