# Supplementary material for: Integrative analyses of genes related to femoral head osteonecrosis: an umbrella review of systematic reviews and meta-analyses of observational studies
Source: J Orthop Surg Res. 2022 Mar 28;17:182. doi: 10.1186/s13018-022-03079-4 (PMC8961967; doi:10.1186/s13018-022-03079-4)
Supplement: Supplementary file 1 — Additional file 1. Table S1: Excluded publications with reasons. [file 13018_2022_3079_MOESM1_ESM.docx]

**Table S1.** Excluded publications with reasons

| **References** | **Reasons for exclusion** | |
| --- | --- | --- |
| Xianyong Yin et al.^1^ | | Not FHON associated article. |
| B.V.K.S. Lakkakula et al.^2^ | | Not FHON associated article. |
| Yangquan Hao et al.^3^ | | No p-value reported. |
| Fa-Qi Guo et al.^4^ | | No statistical calculation reported. |
| Jan S. Moreb et al.^5^ | | Not FHON associated article. |
| Zheng Zeng et al.^6^ | | Duplicated article. |
| Seth E. Karol et al.^7^ | | Insufficient heterogeneity value. (I^2^) |
| Z.C. Zhou et al.^8^ | | Duplicated article. |
| Seth E. Karol et al.^9^ | | Not FHON associated article. |
| Wei Chai et al.^10^ | | No significant results. |
| Patarawan Woratanarat et al.^11^ | | Not FHON associated article. |
| Xiao-Nan Liang et al.^12^ | | Duplicated article. |
| Xi-fu Shang et al.^13^ | | No significant results. |
| Mohammad R. Sobhan et al.^14^ | | Duplicated article. |
| Yu Zhang et al.^15^ | | Duplicated article. |
| Benjie Wang et al.^16^ | | Duplicated article. |

Abbreviations: FHON, Femoral Head Osteonecrosis

1. Yin X, Kim K, Suetsugu H, et al. Meta-analysis of 208370 East Asians identifies 113 susceptibility loci for systemic lupus erythematosus. *Ann Rheum Dis*. 2021;80(5):632-640. doi:10.1136/annrheumdis-2020-219209

2. Lakkakula BVKS. Association between MTHFR 677C>T polymorphism and vascular complications in sickle cell disease: A meta-analysis. *Transfus Clin Biol*. 2019;26(4):284-288. doi:10.1016/j.tracli.2019.01.003

3. Hao Y, Guo H, Xu Z, et al. The relationship between apolipoprotein genes polymorphisms and susceptibility to osteonecrosis of the femoral head: a meta-analysis. *Lipids Health Dis*. 2018;17(1):192. doi:10.1186/s12944-018-0827-0

4. Guo F-Q, Deng M. Correlation Between Steroid-Induced Osteonecrosis of The Femoral Head and Hepatic CYP3A Activity: A Systematic Review and Meta-Analysis. *J Invest Surg*. 2019;32(2):118-126. doi:10.1080/08941939.2017.1385663

5. Yang G, Hamadeh IS, Katz J, et al. *SIRT1/HERC4* Locus Associated With Bisphosphonate-Induced Osteonecrosis of the Jaw: An Exome-Wide Association Analysis: *SIRT1/HERC4* LOCUS ASSOCIATED WITH BP-INDUCED ONJ. *J Bone Miner Res*. 2018;33(1):91-98. doi:10.1002/jbmr.3285

6. Zeng Z, Wang B, Pan H. Relation between osteonecrosis of the femoral head and PAI-1 4G/5G gene polymorphism: a meta-analysis. :6.

7. Karol SE, Jr LAM, Yang W, et al. Genetic risk factors for the development of osteonecrosis in children under age 10 treated for acute lymphoblastic leukemia. 2016;127(5):8.

8. Zhou ZC, Gu SZ, Wu J, Liang QW. VEGF, eNOS, and ABCB1 genetic polymorphisms may increase the risk of osteonecrosis of the femoral head. *Genet Mol Res*. 2015;14(4):13688-13698. doi:10.4238/2015.October.28.31

9. Karol SE, Yang W, Van Driest SL, et al. Genetics of glucocorticoid-associated osteonecrosis in children with acute lymphoblastic leukemia. *Blood*. 2015;126(15):1770-1776. doi:10.1182/blood-2015-05-643601

10. Chai W, Zhang Z, Ni M, et al. Genetic Association between Methylenetetrahydrofolate Reductase Gene Polymorphism and Risk of Osteonecrosis of the Femoral Head. *BioMed Res Int*. 2015;2015:1-6. doi:10.1155/2015/196495

11. Woratanarat P, Thaveeratitharm C, Woratanarat T, Angsanuntsukh C, Attia J, Thakkinstian A. Meta-analysis of hypercoagulability genetic polymorphisms in perthes disease: META-ANALYSIS OF POLYMORPHISMS IN PERTHES. *J Orthop Res*. 2014;32(1):1-7. doi:10.1002/jor.22473

12. Liang X-N, Xie L, Cheng J-W, et al. Association between PAI-1 4G/5G Polymorphisms and osteonecrosis of femoral head: A Meta-analysis. *Thromb Res*. 2013;132(2):158-163. doi:10.1016/j.thromres.2013.06.023

13. Shang X, Su H, Chang W, Wang C, Han Q, Xu Z. Association between MTHFR C677T polymorphism and osteonecrosis of the femoral head: a meta-analysis. *Mol Biol Rep*. 2012;39(6):7089-7094. doi:10.1007/s11033-012-1540-0

14. Sobhan MR, Mahdinezhad-Yazdi M, Moghimi M, et al. Plasminogen Activator Inhibitor-1 4G/5G Polymorphism Contributes to Osteonecrosis of the Femoral Head Susceptibility: Evidence from a Systematic Review and Meta-analysis. . *NUMBER*. 2018;6(6):10.

15. Zhang Y, Xie H, Zhao D, Wang B, Yang L, Meng Q. Association of ABCB1 C3435T polymorphism with the susceptibility to osteonecrosis of the femoral head: A meta-analysis. *Medicine (Baltimore)*. 2017;96(20):e6049. doi:10.1097/MD.0000000000006049

16. Wang B. ABCB1 Gene Polymorphisms and Glucocorticoid-Induced Avascular Necrosis of the Femoral Head Susceptibility: A Meta-Analysis. *Med Sci Monit*. 2014;20:2811-2816. doi:10.12659/MSM.891286
